# Medical Specialty Choice and Related Factors of Brazilian Medical Students and Recent Doctors

**DOI:** 10.1371/journal.pone.0133585

**Published:** 2015-07-24

**Authors:** Ligia Correia Lima de Souza, Vitor R. R. Mendonça, Gabriela B. C. Garcia, Ediele C. Brandão, Manoel Barral-Netto

**Affiliations:** 1 Centro de Pesquisas Gonçalo Moniz, Fundação Oswaldo Cruz (FIOCRUZ), Salvador, Brazil; 2 Faculdade de Medicina, Universidade Federal da Bahia, Salvador, Brazil; 3 Instituto de Biologia, Universidade Federal da Bahia, Salvador, Brazil; 4 Fundação Técnico Educacional Souza Marques, Rio de Janeiro, Brazil; 5 Instituto Nacional de Ciência e Tecnologia em Investigação em Imunologia (iii-INCT), São Paulo, Brazil; University of Minho, PORTUGAL

## Abstract

**Background:**

Choosing a medical specialty is an important, complex, and not fully understood process. The present study investigated the factors that are related to choosing and rejecting medical specialties in a group of students and recent medical doctors.

**Methodology and Findings:**

A cross-sectional survey of 1,223 medical students and doctors was performed in Brazil in 2012. A standardized literature-based questionnaire was applied that gathered preferable or rejected specialties, and asked questions about extracurricular experiences and the influence of 14 factors on a Likert-type scale from 0 to 4. Specialties were grouped according to lifestyle categories: controllable and uncontrollable, which were subdivided into primary care, internal medicine, and surgical specialties. Notably, the time period of rejection was usually earlier than the time period of intended choice (*p* < 0.0001, χ^2^ = 107.2). The choice mainly occurred during the internship period in medical school (*n* = 466; 38.7%). An overall large frequency of participation in extracurricular activities was observed (*n* = 1,184; 95.8%), which were highly associated with the respective medical area. Orthopedic surgery had the highest correlation with participation in specialty-specific organized groups (OR = 59.9, 95% CI = 21.6-166.3) and psychiatry was correlated with participation in research groups (OR = 18.0, 95% CI = 9.0-36.2). With regard to influential factors in controllable lifestyle specialties, “financial reason” (mean score ± standard deviation: 2.8 ± 1.0; median = 3) and “personal time” (3.1 ± 1.3; median = 4) were important factors. In primary care, these factors were less important (1.7 ± 1.3 and 1.7 ± 1.5, respectively; median = 2 for both), and higher scores were observed for “curricular internship” (3.2 ± 1.1, median = 4) and “social commitment” (2.6 ± 1.3, median = 3).

**Conclusion:**

The present findings provide important insights into developing strategies to stimulate interest in specialties based on the needs of the Brazilian healthcare system.

## Introduction

The choice of a medical specialty has implications for both students and the healthcare system. Particularly in countries with a deficit of medical professionals and problems in the distribution of this workforce, the choice of medical specialties is a central issue in attempts to change this problematic situation. Identifying the reasons and factors that underlie the choice of specialties may provide a better understanding of students’ preferences for a given specialty and may aid the development of intervention strategies (i.e., informational programs and extracurricular activities) according to the necessities of healthcare systems.

Medical students select their specialty through a complex process that is related to individual characteristics (e.g., personality [1–4]), demographic factors [2,5–7], experiences during medical school [2,8,9], socialization with professionals, patients, and other students [10], career features [2,10], and other factors. Notably, an important factor that has a strong relationship with the choice of medical specialties is quality of life, which can usurp the influence of such traditional factors as income and gender differences [11]. Since the 1980s, a trend has been seen in medical students who prioritize such specialties as anesthesiology, dermatology, neurology, ophthalmology, otolaryngology, pathology, psychiatry, radiology, and emergency medicine because of lifestyle issues [12,13]. In a longitudinal study by Dorsey et al. that was conducted between 1996 and 2002, more than 55% of the variability in medical students’ specialty preferences was related to controllable lifestyle factors [12]. The characteristics of controllable lifestyle specialties include an individual’s ability to control the time spent on the job and personal time [12,13]. Nevertheless, the worldwide increase in life expectancy and consequent increase in chronic diseases require more primary care (i.e., an uncontrollable lifestyle specialty) physicians to support and prevent diseases [2,14]. However, a decrease has been observed in students’ interest in pursuing uncontrollable lifestyle specialties, which include general specialties, such as primary care (family medicine), obstetrics/gynecology, pediatrics, internal medicine, and surgical specialties [2,12,15,16].

Patients with complex problems, the time spent on the job, physician salaries, medical technology innovations, and increased demands for specialized care are some of the factors that may be associated with rejecting primary care as a specialty [17,18]. The preference for surgical specialties is also likely to decrease, particularly general surgery, mainly because of the long work hours and difficult lifestyle during residency [19]. Notably, specialty preferences might vary according to geographic and culture factors. In New Zealand, general practice (i.e., family medicine) is the third most popular career choice in junior doctors [20]. In contrast, the situation in Brazil is marked by a low interest in general practice (including primary care), which impairs the medical needs for social programs from the universal Brazilian healthcare system [21–23].

The aim of the present study was to evaluate the factors that are related to intention in choosing and rejecting medical specialties and the timing of these decisions during medical school in a large group of students and recent medical doctors in Brazil. Understanding the principal factors that underlie the choice of medical specialties may provide insights into how to increase the preference for often-rejected, albeit essential, medical specialties.

## Methods

### Study design and participants

This was an exploratory cross-sectional study about factors that are related to the choice of specialty in medical students and doctors in two large cities in Brazil (Salvador and Rio de Janeiro) in 2012. These cities represent two of five different regions of the country (northeast and southeast Brazil), and this study covered 20.3% of the medical schools in Brazil (40 of 197 medical schools had at least one participant enrolled herein) [24]. A non-validated literature-based questionnaire was applied in places that had a large circulation of students and medical doctors, such as medical schools, university hospitals, and preparatory course locations for residency programs. The questionnaire was applied anonymously only for internship medical students (i.e., students in their last 2 years of medical school) and physicians who would undergo residency program exams in 2013. These groups of participants were selected because they had a higher probability to have already chosen their specialties with some degree of certainty.

Medical courses in Brazil last for 6 years. The first 4 years are intended to teach theory and practice, and the final 2 years are devoted to supervised practice in internal medicine, surgical areas, pediatrics, family medicine, and obstetrics/gynecology. Participation in the study was voluntary, and the data were reported anonymously.

### Questionnaire

The questionnaire (see S1 Appendix) was based on common factors that may influence the choice of specialty and were explored in previous studies [3,8,10,25–28]. The questionnaire comprised three sections. The first section included demographic data (medical school, gender, marital status, age, city of origin, birthplace, parents’ highest level of education, and parents’ specialty if they were medical doctors). The second section covered extracurricular experiences during medical school (extracurricular internships, participation in research groups, undergraduate teaching assistantship, student activism, and specialty-specific organized groups, such as associations of medical students who are supervised by medical doctors for conducting educational, healthcare, and research activities in a specific medical specialty). The third section consisted of 14 factors that may influence the choice of specialty: “perceived ability” (preference for a set of skills or abilities that are characteristic of the specialty), “way of work,” “autonomy,” “variety of medical problems,” “curricular internship,” “role models,” “financial reason,” “academic experience in this specialty,” “personal time,” “social commitment,” “prestige of specialty,” “residency time,” “research opportunity,” and “family influence.” These factors were evaluated on a 5-point Likert-type scale (0 = no influence, 4 = maximal influence) and classified into two groups: low influence (0 to 2) and high influence (3 to 4). Additionally, the questionnaire inquired about three self-declared options for specialty choices and rejections and the undergraduate period during which the first chosen and rejected options were done.

An initial pilot study was performed with 149 medical students and doctors to improve the questionnaire, but no major alterations were found to be necessary (those questionnaires were also included in the study). All of the participants signed an informed consent form, and the Centro de Pesquisas Gonçalo Moniz—Fundação Oswaldo Cruz -Bahia (CPqGM/FIOCRUZ-BA) Institutional Review Board approved the study (registration no. 225/2012).

### Specialty classification

Specialties in the questionnaire had self-declared options that were divided into two basic groups: controllable and uncontrollable lifestyles. The controllable lifestyle group consisted of anesthesiology, dermatology, neurology, ophthalmology, otolaryngology, pathology, psychiatry, and radiology, as established by Schwartz et al. [13]. In the present study, emergency medicine was excluded from this classification because it is a new field of medical residency that has not yet been consolidated in Brazil [29]. The uncontrollable lifestyle group was subdivided into primary care, internal medicine, and surgical specialties. Primary care comprised family practice, obstetrics/gynecology, pediatrics, and general internal medicine. Internal medicine represented a general specialty and subspecialties classified according to the American College of Physicians [30]. Additionally, surgical specialties included general surgery, neurosurgery, plastic and reconstructive surgery, thoracic surgery, cardiovascular surgery, vascular surgery, urological surgery, hand surgery, head and neck surgery, digestive surgery, surgical oncology, and orthopedic surgery.

### Data analysis

The categorical variables (time of choice and rejection, demographic data [except age], extracurricular experiences, and influence factors based on the Likert-type scale classification [low and high influences]) were compared using the *χ* ^*2*^ test or Fisher’s exact test in 2 × 2 contingency tables along with the relevant odds ratio (OR) and 95% confidence interval (CI) according to the data distribution. The quantitative data were tested for a Gaussian distribution using the D’Agostino and Pearson omnibus normality test (a normal distribution was not found). Differences in ordinal variables (age) between groups were evaluated using the Mann-Whitney test. The Bonferroni correction was used in order to avoid the error type I in tests related to the groups of intended specialty; thus, *p* <0.0019 (considering 26 the number of hypothesis which tested the influence of each variable bellow in the specialty intention of choice: medical school type, gender, age, city of origin, mother and father highest level of education, and parents’ specialty if they were medical doctors, extracurricular internships, participation in research groups, undergraduate teaching assistantship, student activism, and specialty-specific organized groups and “perceived ability”, “way of work,” “autonomy,” “variety of medical problems,” “curricular internship,” “role models,” “financial reason,” “academic experience in this specialty,” “personal time,” “social commitment,” “prestige of specialty,” “residency time,” “research opportunity,” and “family influence” [It does not include the time of choice analysis]) were considered statistically significant. In all other tests the values of *p* < 0.05 were considered statistically significant. The statistical analyses were performed using Prism 5.0b software (GraphPad, San Diego, CA, USA).

## Results

### Baseline characteristics

A total of 1,547 questionnaires were distributed, with a response rate of 79.2% (*n* = 1,225). Two of the 1,225 questionnaires were excluded because less than 25% of the questions were answered. The demographic characteristics of the remaining 1,223 respondents are presented in Table 1. Interestingly, the number of participants who had at least one parent who was a physician was high (*n* = 301; 24.6%), with high concordance between the mothers’ (35.9%) and fathers’ (34.5%) medical specialties and the participants’ intended specialty.

**Table 1:** Demographic characteristics of the individuals enrolled in the study.

Demographic characteristics	No.	%
**Age in years (median)**	24	NA
**Female**	764	62.5
**Medical student in internship period**	857	70.1
**City of origin before medical school with > 500,000 inhabitants**	884	72.3
**Private medical school**	691	56.5
**Mother's education**		
**University**	888	72.6
**High school**	292	23.9
**Elementary school**	40	3.3
**Illiterate**	3	0.2
**Father's education**		
**University**	883	72.4
**High school**	275	22.6
**Elementary school**	59	4.8
**Illiterate**	3	0.2
**At least one parent is a medical doctor**	301	24.6

NA, not applicable.

### Chosen and rejected specialties

A total of 2,590 intentions of choice and 3,014 rejections were made among the six self-declared options among the 1,223 participants (up to three options for choice and three for rejection per participant).

Controllable lifestyle specialties were responsible for 32.5% (*n* = 396) of the first choices. Anesthesiology had the highest frequency of choice in this group (8.9%; *n* = 108; Table 2). Specialties with a controllable lifestyle comprised 27.4% (*n* = 827) of the rejections, and the most rejected specialty in this group was psychiatry (6.7%; *n* = 201; Table 2). In the uncontrollable lifestyle group, the primary care subgroup comprised 26.7% (*n* = 326) of the first choices and more than one-third of all of the rejections (35.1%; *n* = 1057; Table 2). Pediatrics comprised the majority of the first choices (10.5%; *n* = 128; Table 2), and obstetrics/gynecology was the most rejected specialty in this subgroup (14.5%; *n* = 437; Table 2). The internal medicine subgroup comprised 25.3% (*n* = 309) of the first choices and only 16.6% (*n* = 499) of the rejections (Table 2). Surgical specialties comprised 22.3% (*n* = 272) of the first choices and 23.2% (*n* = 701) of the rejections (Table 2).

**Table 2:** Choice and rejection of medical specialties classified by groups of specialties.

Group	Subgroup	Specialty	First Choice No. (%)	All Choices No. (%)	Rejection No. (%)
**Controllable lifestyle**	-	Anesthesiology	108 (8.9)	189 (7.3)	39 (1.3)
	-	Dermatology	70 (5.8)	117 (4.5)	161 (5.3)
	-	Neurology	27 (2.2)	57 (2.2)	97 (3.2)
	-	Ophthalmology	80 (6.6)	137 (5.3)	140 (4.7)
	-	Otolaryngology	38 (3.1)	82 (3.2)	51 (1.7)
	-	Pathology	5 (0.4)	11 (0.4)	77 (2.6)
	-	Psychiatry	21 (1.7)	50 (1.9)	201 (6.7)
	-	Radiology	47 (3.9)	121 (4.7)	61 (2.0)
**Uncontrollable lifestyle**	PC	Family Practice	15 (1.2)	48 (1.9)	79 (2.6)
	PC	Obstetrics and Gynecology	79 (6.5)	143 (5.5)	437 (14.5)
	PC	Pediatrics	128 (10.5)	228 (8.8)	398 (13.2)
	PC/IM	General Internal Medicine	104 (8.5)	225 (8.7)	143 (4.8)
	IM^§^	Internal Medicine (not general)	205 (16.8)	526 (20.3)	356 (11.8)
	SS	Orthopedic surgery	52 (4.3)	86 (3.3)	233 (7.7)
	SS	Surgery^†^	220 (18.0)	443 (17.1)	468 (15.5)
	-	Intensive Care Medicine	5 (0.4)	66 (2.5)	9 (0.3)
**Not applied**		Others	15 (1.2)	61 (2.4)	64 (2.1)
		Total	1219	2590	3014

PC, primary care; IM, internal medicine (general); SS, surgical specialties. ^§^Internal medicine, including allergy/immunology, cardiology, endocrinology, hematology, gastroenterology, oncology, infectious disease, pulmonary diseases, nephrology, rheumatology, and geriatric medicine. ^†^Surgery, including general surgery, neurological surgery, plastic and reconstructive surgery, thoracic surgery, cardiovascular surgery, general vascular surgery, urological surgery, hand surgery, head and neck surgery, digestive surgery, and surgical oncology.

**Fig 1:**
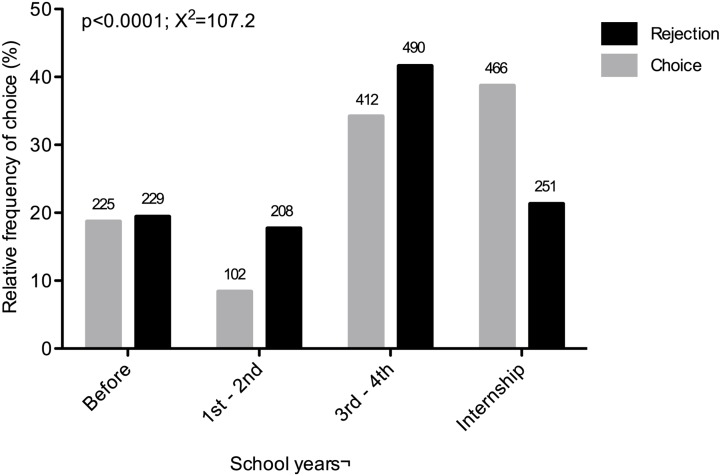
Period of choice and rejection of medical specialties. The figure presents the temporal distribution (in school years) of choice and rejection of self-declared first-option specialties. The gray columns represent the choice of specialty. The black columns represent the rejection of specialties. The numbers above the columns represent the absolute frequency of choice/rejection in the corresponding period.

Notably, the time period of rejection was usually earlier than the time period of choice (*p* < 0.0001; *χ* ^*2*^ = 107.2; Fig 1). Choice mainly occurred during the internship period in medical school (*n* = 466; 38.7%). Interestingly, 18.7% of the participants already chose their specialty before medical school and remained with this choice throughout their studies. The predominant rejection period was between the third and fourth years of medical school (*n* = 490; 41.6%; Fig 1). The period of intended choice was different according to specialty group (S1A Fig).

Both the primary care and surgical specialties were more likely to be chosen before entering medical school (primary care group: *n* = 77, 24.1% of choices, *p* = 0.0056, OR = 1.6, 95% CI = 1.2–2.2; surgical specialty group: *n* = 64; 23.6% of choices, *p* = 0.0212, OR = 1.5, 95% CI = 1.1–2.1; S1B Fig). Afterward, these two groups diverged, in which surgical specialties were mainly chosen between the third and fourth years, and primary care was chosen during internships.

The internal medicine specialty and controllable lifestyle had a lower chance of being chosen before medical school (internal medicine group: *n* = 40, 13.1% of choices, *p* = 0.0038, OR = 0.6, 95% CI = 0.4–0.8; controllable lifestyle group: *n* = 51, 13.0% of choices, *p* = 0.0005, OR = 0.6, 95% CI = 0.4–0.8; S1B Fig). Controllable lifestyle specialties were also more likely to be chosen during the internship period (controllable lifestyle group: *n* = 197, 59.4% of choices, *p* < 0.0001, OR = 2.1, 95% CI = 1.6–2.6; S1B Fig).

### Specialties and demographic factors

Females chose specialties that are likely associated with working in “primary care” (OR = 3.8, 95% CI = 2.8–5.2, *p* < 0.0001) as a first choice, whereas males chose surgical specialties (OR = 3.7, 95% CI = 2.8–4.9; *p* < 0.0001; S1 Table).

No differences were found in other demographic variables (age, city of origin, parents’ education, parents medical doctors) and the probability to choose specialty groups (S1 Table).

### Extracurricular experience during medical school

Overall, the majority of the participants engaged in at least one extracurricular activity during medical school (*n* = 1,184; 95.8%) and most of them participated in extracurricular internships (*n* = 1093; 89.4%), research groups (*n* = 716; 58.6%), specialty-specific organized groups (*n* = 814; 66.6%), or undergraduate teaching assistantships (*n* = 755; 61.7%). Only 11.4% (*n* = 140) of the subjects participated in student activism activities.

Participation in extracurricular activities was more frequently observed in those who attended public universities compared with private universities (extracurricular internship, *p* = 0.0225; research group, *p* < 0.0001; specialty-specific organized groups, *p* = 0.0009; undergraduate teaching assistantship, *p* < 0.0001; student activism, *p* < 0.0001).

Extracurricular experiences had an important influence on the medical specialty chosen in one of three self-declared options of intended choice (Table 3). Ophthalmology (OR = 27.2, 95% CI = 10.6–69.9), dermatology (OR = 13.9, 95% CI = 5.7–33.8), obstetrics/gynecology (OR = 15.6, 95% CI = 9.4–25.8), internal medicine and subspecialties (OR = 2.4, 95% CI = 1.8–3.1), orthopedic surgery (OR = 59.9, 95% CI = 21.6–166.3), and surgery (OR = 6.0, 95% CI = 4.3–8.2) had higher correlations with participation in specialty-specific organized groups in their respective area. Research was the most influential extracurricular activity in psychiatry (OR = 18.0, 95% CI = 9.0–36.2; Table 3). For neurology (OR = 19.2, 95% CI = 8.6–42.9), pediatrics (OR = 12.5, 95% CI = 8.5–18.3), and cardiology (OR = 15.6, 95% CI = 8.6–28.4), the most related extracurricular activity was extracurricular internship in their respective medical area.

**Table 3:** Extracurricular activity during medical school and probability of choosing a corresponding specialty (between one of three self-declared options).

Group	Specialty	Extracurricular internship OR (95% CI)*	Medical student study groups OR (95% CI)*	Research OR (95% CI)*
**Controllable lifestyle**	Dermatology	Not reliable**	13.9 (5.7–33.8)	8.5 (3.6–20.2)
	Neurology	19.2 (8.6–42.9)	13.0 (6.6–25.4)	15.2 (6.8–34.2)
	Ophthalmology	Not reliable**	27.2 (10.6–69.9)	Not reliable
	Psychiatry	Not reliable**	13.2 (5.1–34.2)	18.0 (9.0–36.2)
**Uncontrollable Lifestyle**	Obstetrics and gynecology	12.2 (7.7–19.3)	15.6 (9.4–25.8)	8.9 (3.7–21.4)
	Pediatrics	12.5 (8.5–18.3)	9.5 (6.0–15.0)	4.7 (3.1–7.2)
	Internal medicine^§^	2.1 (1.6–2.8)	2.4 (1.8–3.1)	2.1 (1.6–2.8)
	Cardiology	15.6 (8.6–28.4)	8.0 (5.2–12.3)	8.4 (4.7–15.0)
	Orthopedic surgery	30.5 (16.3–57.0)	59.9 (21.6–166.3)	Not reliable**
	Surgical specialties^†^	5.7 (4.2–7.7)	6.0 (4.3–8.2)	3.7 (2.4–5.5)

*p < 0.0019 (Fisher’s test).

**Unreliable data; answers to the question had an absolute frequency of less than 15.

^§^Internal medicine, including general internal medicine, allergy/immunology, cardiology, endocrinology, hematology, gastroenterology, oncology, infectious disease, pulmonary diseases, nephrology, rheumatology, and geriatric medicine.

^†^Surgical specialties, including general surgery, neurological surgery, plastic and reconstructive surgery, thoracic surgery, cardiovascular surgery, general vascular surgery, urological surgery, hand surgery, head and neck surgery, digestive surgery, surgical oncology, and orthopedic surgery.

### Factors related to intended choice of specialty

Considering all of the intended choices of specialties, on a scale from 0 to 4, the most influential factors of the 14 factors evaluated was “perceived ability” and “way of work” (median = 4 for both; Table 4). “Family influence” and “research opportunity” had the lowest scores (median = 0 and 1, respectively; Table 4) in the entire sample.

**Table 4:** Influential factors of intended choice (first choice) by groups of medical specialties. The numbers represent the median and interquartile range for factors in each group of medical specialties according to a Likert scale.

Factors	Median (interquartile range)				
	Controllable lifestyle	Uncontrollable lifestyle—PC	Uncontrollable lifestyle—SS	Uncontrollable lifestyle-IM	All
**Perceived ability**	3 (3–4)	4 (3–4)	4 (3–4)	4 (3–4)	4 (3–4)
**Way of wok**	4 (3–4)*	3.5 (3–4)*	4 (3–4)	4 (3–4)	4 (3–4)
**Autonomy**	3 (2–4)*	2 (1–3)*	3 (2–4)	3 (2–4)	3 (2–4)
**Variety of medical problems**	3 (2–4)	3 (2–4)	3 (2–4)	3 (2–4)	3 (2–4)
**Internship (curricular)**	2 (1–4)*	4 (3–4)*	3 (2–4)	3 (2–4)	3 (2–4)
**Role models**	3 (1–4)	3 (2–4)	3 (1–4)	3 (2–4)*	3 (2–4)
**Financial reason**	3 (2–3)*	2 (0–3)*	3 (2–3)*	2 (1–3)*	3 (2–3)
**Academic experience**	2 (1–3)*	3 (2–4)	3 (2–4)	2 (1–3)	2 (1–4)
**Personal time**	4 (2–4)*	2 (0–3)*	1 (0–2)*	2 (1–3)	2 (1–4)
**Social commitment**	2 (1–3)*	3 (2–4)*	2 (1–3)	3 (2–3)*	2 (1–3)
**Prestige of specialty**	2 (1–3)*	1 (0–2)*	2 (1–3)*	2 (1–3)	2 (1–3)
**Residency time**	2 (0–3)*	1 (0–2)	0 (0–2)*	0 (0–2)*	1 (0–3)
**Research opportunity**	1 (0–2)	0 (0–2)	1 (0–2)	1 (0–3)	1 (0–2)
**Family influence**	0 (0–2)	0 (0–1)	0 (0–1)	0 (0–2)	0 (0–2)

*p < 0.0019, comparison between the selected group and the rest of the sample considering two groups: low influence (0–2 Likert scale) and high influence (3–4 Likert scale; Fisher test). PC, primary care; SS, surgical specialties; IM, internal medicine.

**Fig 2:**
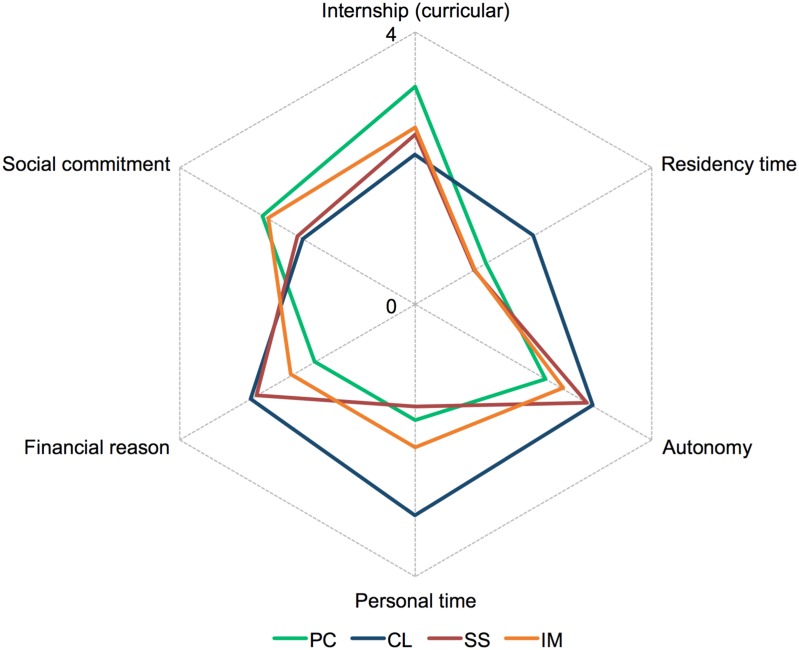
Influential factors of choice (first choice) by groups of medical specialties. The graphic represents the distribution of the mean influence score of six choice factors (selected from 14, according to arithmetic mean difference) in groups of medical specialties. PC, primary care; CL, controllable lifestyle; SS, surgical specialties; IM, internal medicine and subspecialties.

The gradation of influential factors varied according to the group of specialties (Fig 2, spider graph inputted with mean values) and were classified into two groups: low influence (0 to 2) and high influence (3 to 4). “Autonomy” (mean ± standard deviation: 3.0 ± 1.2; 78.3% with high influence, *p* < 0.0001), “financial reason” (2.8 ± 1.0; 67.3% with high influence, *p* < 0.0001), and “personal time” (3.1 ± 1.3; 74.6% with high influence, *p* < 0.0001) were important factors for choosing controllable lifestyle specialties. The primary care group was largely influenced by “curricular internship” (3.2 ± 1.1; 76.7% with high influence, *p* < 0.0001) and “social commitment” (2.6 ± 1.3; 59.3% with high influence, *p* < 0.0001) but less influenced by “financial reason” (1.7 ± 1.3; 68.2% with low influence, *p* < 0.0001) and “personal time” (1.7 ± 1.5; 66.7% with low influence, *p* < 0.0001). Surgical specialties had a low influence from “personal time” (1.5 ± 1.4; 77% with low influence, *p* < 0.0001) and “residency time” (1.0 ± 1.3; 85.9% with low influence, *p* < 0.0001) but a high influence from “financial reason” (2.7 ± 1.1; 61.9% with high influence, *p* < 0.0014). Internal medicine had a low influence from “residency time” (1.0 ± 1.2; 87.9% with low influence, *p* < 0.0001) and a high influence from “social commitment” (2.5 ± 1.2; 54.6% with high influence, *p* < 0.0001).

## Discussion

The factors that underlie the choice or rejection of a medical specialty is largely unexplored in Brazil, a country with a deficiency of medical doctors. This was a pioneering large-sample study that explored the time of probable intention and rejection of medical specialties during medical school. Interestingly, we found that the time of rejection of a medical specialty generally occurred before the intention of choice during medical school. Furthermore, we found that a high percentage of the respondents had parents who were doctors, and their specialties were highly correlated with their children’s specialty intention. The present report demonstrates that the factors that underlie specialty choice in Brazil are similar to the profiles in other countries, thus confirming the stratification of influential factors according to groups of medical specialties (i.e., controllable *vs*. uncontrollable lifestyle specialties). Moreover, associations between extracurricular experiences during medical school and the intention of pursuing a specific medical area were observed in this study.

The present report reveals that the decision to reject a specialty usually preceded the intention of choice (or occurred at the same time), which may reflect exposure to medical practices that subsequently influences preferences for different specialties. Overall, more than one-third of first choices occurred during the internship stage, possibly reflecting practical learning experiences that influence medical careers. Notably, a high frequency of controllable lifestyle specialty choices also occurred during the internship period, concomitant with a decrease in the choice of surgical specialties. This decrease in surgical choice may reflect the perception of a poor lifestyle associated with a surgical career that is observed during practical rotations in medical school [13,19]. Maiorova et al. reported that medical students use clinical rotations to learn more about the practical side of medicine and discover the advantages and disadvantages of each specialty [31]. However, the choice of specialty can be unstable, even during postgraduate years, and the definitive choice of medical specialty may occur after completing medical school [32]. Surprisingly, the main rejection period was between the third and fourth years of medical courses, a period of transition from basic and general disciplines to initial contact with medical specialties in Brazil. Considering these data together, medical students initially usually excluded some specialties, and they made their intentions of choice during clinical rotations.

Surprisingly, nearly a quarter of the participants in the present study had at least one parent who was a doctor, and their specialties had high concordance with one of the three intentions of specialty choice. This concordance may be a consequence of family influence and admiration of their parent’s specialty. Similarly, having a parent who is a general practitioner is strongly correlated with a career intention to pursue general practice [33]. In the present study, as in previous reports [34], female students preferred primary care specialties. An increasing number of women are pursuing medical careers in both Brazil and other countries [34,35], and an increase in the preference for primary care specialties would be expected. Nevertheless, such an increase has not been observed in Brazil [36], which may be counterbalanced by an increase in the preference for controllable lifestyle specialties by both genders [37].

Brazilian medical students usually participate in several extracurricular activities during their course work that may influence their interest in a specific specialty [38,39]. One limitation of the present study was its cross-sectional design, which precludes the determination of causal relationships. With this in mind, participation in extracurricular activities within a specialty was highly correlated with a greater probability of choosing a specialty in the same area in this report, but this may also be a consequence of a choice that was already made by the medical student. Extracurricular activities during medical school can be a great opportunity to learn and practice a specific medical field in parallel with mandatory undergraduate activities [38,39]. In this context, because of the robust association between experience and the chosen specialties, extracurricular activities may be useful to increase students’ attraction to specific areas of medicine. Several studies have reported that experiences in medical school, occupational aspects (such as prestige and income), and individual aspects (such as personal competency, ambitions, work-life balance, and affinities) were the main factors that influence the choice of specialty [3,25,40,41].

The factors that influenced the intention of choice of specialties were different according to groups of specialties, suggesting different motivational profiles. In this study, subjects who chose controllable lifestyle specialties put more value on “personal time,” “financial reason,” “residency time,” and “autonomy” compared with the other groups. These findings are compatible with the perspective that preferences for controllable lifestyle specialties are associated with interest in a stable and secure career, a good quality of life, and better control of the time spent during work [4,13]. In contrast, “personal time” had little influence on the choice of surgical specialties. The perception of a poor lifestyle associated with surgical specialties is an important factor that may be associated with a decrease in their popularity [19]. Furthermore, the internal medicine group was characterized by high scores on “social commitment” and low scores on “financial reason,” a profile that was similar to the primary care group. Despite these similarities, few internal medicine residents (20–25%) planned to pursue careers as general clinicians and instead preferred subspecialty areas [42,43].

In the present study, individuals who pursued primary care specialties gave high scores to “social commitment” and experiences during “internships” and low scores to “financial reason” and “personal time.” This profile of factors that influence the aspiration to pursue primary care specialties is compatible with a more idealistic orientation with less importance placed on social status. However, a decline of idealism has been reported during medical school, which may be linked with increasing disinterest in primary care specialties [44]. A few Brazilian studies investigated medical specialty choice and found that medical students rejected primary care medicine [21,22] for such factors as financial reasons and quality of life [22,41]. Given that the universal Brazilian healthcare system emphasizes preventive medicine, the low interest of students in pursuing primary care specialties is a major national issue. This situation is aggravated by the lack of physicians (national mean of 1.8 professionals per 1000 habitants) and an unequal geographic distribution of this workforce [23,36]. Importantly, a survey of medical residents in the United States reported that internships significantly influence the choice of primary care [45]. Therefore, experiences during internships or increased exposure of medical students to primary care specialties may stimulate interest in these specialties.

Although just two cities in Brazil were studied herein, 20.3% of the medical schools from the country were included, suggesting that this sampling may be representative of Brazilian medical students overall. The participants’ gender (62.5% female) is compatible with the national mean (53.5% female) reported in 2012 [35]. Similarly, the distribution of students according to private and public institutions in the present study (56.5% from private medical schools) was consistent with the national distribution of 58.7% of medical students from private schools reported in 2012 [46].

An important limitation of the present study concerns the questionnaire that was given to individuals who were still in the process of choosing their medical specialties. The questionnaire measured choice preferences at a specific time-point (career aspirations) rather than their actual choice of a medical specialty. Two biases that would influence the present results need to be mentioned. First, 20.8% of individuals did not answer the questionnaire, indicating possible selection bias. Second, the temporal questions that were asked in the questionnaire may have caused recall bias. Another limitation was the classification of specialties according to lifestyle (controllable or uncontrollable, which may create some generalizations that do not necessarily correspond with the realities of local medical practice). Although this classification has been extensively used in studies from other countries (e.g., United States) [13,28], this classification was not previously used in Brazil. However, the profile of factors that influence groups of specialties in Brazil based on lifestyle issues in the present study is similar to profiles from previous studies [12].

The present results suggest that some factors should be further explored to expand medical students’ choices of needed medical specialties based on data from two large Brazilian cities, but the results may not be generalizable to all places. “Perceived ability” and “way of work” had the highest mean scores among all of the factors, and these factors are not amenable to intervention. However, “internship,” “role models,” and “financial reason” also had high mean scores and are amenable to intervention. Therefore, certain strategic interventions may increase the choice of specialties that currently suffer from shortages of professionals in Brazil. We propose the expansion of opportunities during internships, empowerment of role models (e.g., teachers and physicians with better pedagogical capabilities and communication skills), and financial incentives that can be used to increase the preference for some specialties in Brazil, which has also been suggested elsewhere [28,31,47]. Furthermore, strategies can be developed that center on the main moments of choice in each specialty group and may be useful for increasing the preference for these specialties. In such a scenario, critical primary care specialties are likely to be chosen before medical school. Therefore, informational programs for students who want to pursue a career in medicine can be implemented before they enter medical school to inform them about the importance and benefits of such specialties to stimulate interest in these areas.

## Conclusion

Choosing a medical specialty is important for both students and the healthcare system. The present results demonstrated that the intention of choice of medical specialties occurs mainly at the end of medical school, and the rejection of specialties occurs as the beginning of medical school. These time differences vary according to the group of medical specialties. During medical courses, participation in extracurricular activities (e.g., specialty-specific organized groups and undergraduate teaching assistantships) may be used to increase the preference for important and urgently needed specialties. Furthermore, different groups of specialties have different influential factors, suggesting that an increase in the preference for primary care specialties requires greater internship experience. With regard to controllable lifestyles specialties, “financial reason,” “autonomy,” and “personal time” were the most important factors for choosing these specialties. The present results elucidate the factors associated with the intention of choice of medical specialties in two large cities in Brazil and may be useful to stimulate interest in new specialists according to healthcare system needs, although these findings may not be generalizable and applied everywhere.

## Supporting Information

S1 Appendix Questionnaire: Understanding the Factors Underlying Medical Specialty Choice. (PDF)

S1 Fig Differences in the moment of the choice of first-option specialty by groups of medical specialties. **(A)** The distribution of groups of medical specialties (internal medicine, surgical specialties, primary care, and controllable lifestyle) as a percentage of first choice in each period of medical school. **(B)** Odds ratios (95% confidence intervals) of medical specialty group chosen in a specific period of medical school (before medical school [Bef MS], between first and second year [1^st^-2^nd^], between third and fourth year [3^rd^-4^th^], and internship) and the corresponding *p* value.
(EPS)

S1 Table Demographic characteristics of participants classified by groups of specialties. *p < 0.0019 (Fisher test; values specified in the text with OR and CI). PC, primary care; SS, surgical specialties; IM, internal medicine.
(PDF)
