# Supplementary material for: Medical Specialty Choice and Related Factors of Brazilian Medical Students and Recent Doctors
Source: PLoS One. 2015 Jul 24;10(7):e0133585. doi: 10.1371/journal.pone.0133585 (PMC4514603; doi:10.1371/journal.pone.0133585)
Supplement: S1 Appendix — (PDF) [file pone.0133585.s001.pdf]

S1 Appendix. Questionnaire: Understanding the Factors Underlying Medical Specialty Choice.

University:	( )	Medical student (period in school)		Age:		Gender:	( ) M	( )F
	( )	Physician (time since graduation)						

Marital status: ( ) Single ( ) Married ( ) Stable union

Birthplace:

City of origin before medical

Mother's education:	( )	Illiterate	( )	Elementary school	( )	High school	( )	University
Father's education	( )	Illiterate	( )	Elementary school	( )	High school	( )	University

Are your parents physicians? ( ) Yes, both. ( ) Yes, only one of them. ( ) No.

What is their medical specialty? Mother: Father:

What type of school did you go for high school?

( ) Public ( ) Private

Did you participate in medical students' study groups? In which medical specialty?

( )	No	( )	Obstetrics and Gynecology	( )	Orthopedics	( )	Pediatrics
( )	Internal medicine and subspecialties	( )	Surgery and subspecialties	( )	Emergence		

Subspecialty: Other specialty:

Did you participate in extracurricular internships? In which medical specialty?

( )	No	( )	Obstetrics and Gynecology	( )	Orthopedics	( )	Pediatrics
( )	Internal medicine and subspecialties	( )	Surgery and subspecialties	( )	Emergence	( )	Public health

Subspecialty: Other specialty:

Did you participate in research groups? In which medical specialty?

( )	No	( )	Epidemiolog y	( )	Primary care	( )	Pediatrics
( )	Internal medicine and subspecialties	( )	Surgery and subspecialtie s	( )	Emergence	( )	Immunopathology

Subspecialty: Other specialty:

Did you participate in student activisms?

( ) Yes ( ) No

Did you participate in undergraduate teaching assistantship? In which area?

( ) No ( ) Yes, in the area:

Which specialties would you like to pursue?

1st option

2nd option

3rd option

When did you interest in the specialty of choice (1st option)?

( ) Before medical school ( ) 1st - 2nd medical school years ( ) 3rd - 4th medical school years ( ) During internship

Which specialties would you reject (would not pursue) ?

1st option

2nd option

3rd option

When did you reject the rejected specialty (1st option)?

( ) Before medical school ( ) 1st - 2nd medical school years ( ) 3rd - 4th medical school years ( ) During internship

Do you want to work in rural area or small towns after graduated?

( ) Yes ( ) No

Score the influence of the following items between 0 to 4 according to your medical specialty choice.

* 0 is no influence and 4 denotes maximum influence

Financial reason	(0)	(1)	(2)	(3)	(4)
Perceived ability	(0)	(1)	(2)	(3)	(4)
Academic experience	(0)	(1)	(2)	(3)	(4)
Nice internship (curricular) in this specialty	(0)	(1)	(2)	(3)	(4)
Role models	(0)	(1)	(2)	(3)	(4)
Family influence	(0)	(1)	(2)	(3)	(4)
Prestige of specialty	(0)	(1)	(2)	(3)	(4)
Autonomy potential	(0)	(1)	(2)	(3)	(4)
Residency time	(0)	(1)	(2)	(3)	(4)
Personal and family time protection	(0)	(1)	(2)	(3)	(4)
Research opportunity	(0)	(1)	(2)	(3)	(4)
Social commitment	(0)	(1)	(2)	(3)	(4)
Variety of medical problems	(0)	(1)	(2)	(3)	(4)

Way of work	(0)	(1)	(2)	(3)	(4)
