# Supplementary material for: Medical Specialty Choice and Related Factors of Brazilian Medical Students and Recent Doctors
Source: PLoS One. 2015 Jul 24;10(7):e0133585. doi: 10.1371/journal.pone.0133585 (PMC4514603; doi:10.1371/journal.pone.0133585)
Supplement: S1 Table — *p < 0.0019 (Fisher test; values specified in the text with OR and CI). PC, primary care; SS, surgical specialties; IM, internal medicine. (PDF) [file pone.0133585.s003.pdf]

S1 Table. Demographic characteristics of participants classified by groups of specialties.

Demographic characteristics	No. (%)			
	Controllable lifestyle	Uncontrollable lifestyle		
		PC	SS	IM
Age in years, mean ± SD	25.2 (±2.8)	25.0 (±2.8)	25.2 (±2.7)	25.1 (±2.5)
Female	244 (61.6)	268 (82.2)*	104 (38.2)*	202 (65.4)
City of origin before medical school with > 500,000 inhabitants	296 (74.7)	229 (70.2)	198 (72.8)	219 (70.9)
Private medical school	234 (59.1)	175 (53.7)	157 (57.7)	159 (51.5)
Mother's education (university)	286 (72.2)	238 (73.0)	198 (72.8)	219 (70.9)
Father's education (university)	282 (71.2)	237 (72.7)	198 (72.8)	218 (70.6)
At least one parent is a medical doctor	107 (27.0)	73 (22.4)	56 (20.6)	81 (26.2)

*p<0.0019 (Fisher test; values specified in the text with OR and CI). PC, primary care; SS, surgical specialties; IM, internal medicine.
